# First clinical implementation of dynamic tumor-tracking volumetric modulated arc therapy with a gimbal-mounted linac: short delivery time and high precision

**DOI:** 10.1093/jrr/rraf093

**Published:** 2026-03-06

**Authors:** Noriko Kishi, Mitsuhiro Nakamura, Hideaki Hirashima, Takanori Adachi, Masahiro Yoneyama, Kohei Kawata, Yukako Kishigami, Takashi Ogata, Takashi Murakami, Yusuke Iizuka, Hiroshi Seno, Takashi Mizowaki

**Affiliations:** Department of Radiation Oncology and Image-Applied Therapy, Graduate School of Medicine, Kyoto University, 54 Shogoin-Kawahara-cho, Sakyo-ku, Kyoto, 606-8507, Japan; Department of Advanced Medical Physics, Graduate School of Medicine, Kyoto University, 53 Shogoin-Kawahara-cho, Sakyo-ku, Kyoto, 606-8507, Japan; Department of Radiation Oncology and Image-Applied Therapy, Graduate School of Medicine, Kyoto University, 54 Shogoin-Kawahara-cho, Sakyo-ku, Kyoto, 606-8507, Japan; Department of Radiation Oncology and Image-Applied Therapy, Graduate School of Medicine, Kyoto University, 54 Shogoin-Kawahara-cho, Sakyo-ku, Kyoto, 606-8507, Japan; Department of Radiation Oncology and Image-Applied Therapy, Graduate School of Medicine, Kyoto University, 54 Shogoin-Kawahara-cho, Sakyo-ku, Kyoto, 606-8507, Japan; Division of Clinical Radiology Service, Kyoto University Hospital, 53 Shogoin-Kawahara-cho, Sakyo-ku, Kyoto, 606-8507, Japan; Department of Radiation Oncology and Image-Applied Therapy, Graduate School of Medicine, Kyoto University, 54 Shogoin-Kawahara-cho, Sakyo-ku, Kyoto, 606-8507, Japan; Department of Radiation Oncology and Image-Applied Therapy, Graduate School of Medicine, Kyoto University, 54 Shogoin-Kawahara-cho, Sakyo-ku, Kyoto, 606-8507, Japan; Department of Radiation Oncology and Image-Applied Therapy, Graduate School of Medicine, Kyoto University, 54 Shogoin-Kawahara-cho, Sakyo-ku, Kyoto, 606-8507, Japan; Department of Radiation Oncology and Image-Applied Therapy, Graduate School of Medicine, Kyoto University, 54 Shogoin-Kawahara-cho, Sakyo-ku, Kyoto, 606-8507, Japan; Department of Gastroenterology and Hepatology, Graduate School of Medicine, Kyoto University, 54 Shogoin-Kawahara-cho, Sakyo-ku, Kyoto, 606-8507, Japan; Department of Radiation Oncology and Image-Applied Therapy, Graduate School of Medicine, Kyoto University, 54 Shogoin-Kawahara-cho, Sakyo-ku, Kyoto, 606-8507, Japan

**Keywords:** dynamic tumor tracking, volumetric modulated arc therapy, gimbal-mounted linac, stereotactic body radiotherapy

## Abstract

Dynamic tumor tracking (DTT) is an effective respiratory motion management strategy for thoracic and abdominal tumors and enables treatment delivery under free breathing using gimbal-mounted linac, without requiring breath-holding. To date, however, clinical implementations have used three-dimensional conformal radiotherapy (3D-CRT) or fixed-field intensity-modulated radiotherapy (IMRT), and integration with volumetric modulated arc therapy (VMAT) has been anticipated to shorten treatment time while improving dose distribution. This short communication reports the first clinical implementation of stereotactic body radiotherapy with DTT-VMAT using a novel gimbal-mounted linac. Planned treatment using DTT-VMAT for liver tumor over five treatment days was delivered successfully, with a mean treatment time of 23.2 min (range, 12–33 min), shorter than previously reported gimbal-based DTT with 3D-CRT or fixed-field IMRT. The average 3D tracking error was 2.4 ± 1.8 mm overall, comprising 0.3 ± 1.0 mm laterally, −1.5 ± 2.2 mm longitudinally and − 0.1 ± 1.0 mm vertically, maintaining tracking performance comparable to earlier systems. No updates to the four-dimensional model were required during beam delivery. This introduction of novel gimbaled-based DTT-VMAT approach is advantageous among available respiratory motion management techniques, as it shortens treatment time, mitigates baseline drift and improves patient compliance.

## INTRODUCTION

Stereotactic body radiotherapy is widely used for the treatment of small thoracic and upper-abdominal tumors, such as early-stage lung cancer and hepatocellular carcinoma (HCC). SBRT enables delivery of a highly conformal dose to a small target volume with a steep dose gradient, thereby minimizing the dose to surrounding organs at risk. In particular, tumors located in the lower lung lobes and liver can exhibit considerable respiratory motion, and robust motion management is essential for safe and effective SBRT in these regions to maintain target coverage and organ-at-risk sparing [1].

Effective respiratory motion management strategies for SBRT in the lower lung lobes and liver include breath-hold, respiratory gating and dynamic tumor tracking (DTT), each with inherent advantages and disadvantages. Breath-hold and gating can substantially reduce motion but often prolong treatment time and may be difficult to maintain in patients with deteriorated pulmonary function [2]. By contrast, DTT allows irradiation under free breathing and can improve patient comfort. In DTT-SBRT, fiducial markers are implanted adjacent to the tumor via a percutaneous or transendoscopic approach, serving as a surrogate for real-time tumor positioning. Tumor motion is correlated with abdominal wall displacement, which enables the construction of a four-dimensional (4D) model for dynamic tracking [3]. Historically, DTT-SBRT has been delivered using three-dimensional conformal radiotherapy (3D-CRT) or fixed-field intensity-modulated radiotherapy (IMRT) [4–8], which has improved dose distribution drastically but tends to require prolonged beam-on and in-room times.

To overcome the long treatment times associated with fixed-field DTT-IMRT and to further streamline free-breathing treatment, recent advancements have enabled multi-leaf collimator-based real-time tumor tracking with volumetric modulated arc therapy (VMAT) [9]. However, in such systems, both tracking and intensity modulation rely on the MLCs, and this technique is limited by leaf length and maximum leaf motion speed, with an average treatment time of approximately 90 min, which is clinically impractical and potentially intolerable for patients who must maintain an arm-raised position during beam delivery.

A novel gimbal-mounted linac developed in Japan addresses this problem by separating tracking from intensity modulation, enabling DTT-VMAT with much shorter treatment times and potentially making free-breathing SBRT more feasible than other motion-management strategies. Here, we report the first clinical application of SBRT with DTT-VMAT using this novel gimbal-mounted linac for the treatment of HCC at our institution.

## MATERIALS AND METHODS

The novel gimbal-mounted linac, the OXRAY system (Hitachi High-Tech Co., Ltd., Tokyo, Japan), is the successor to the Japan-developed Vero4DRT system (Hitachi High-Tech Co., Ltd., Tokyo, Japan). The Vero4DRT had several limitations, including a relatively small field size, availability of only 6-MV beams with a flattening filter, 5-mm MLCs, and, for DTT, compatibility only with 3D-CRT or IMRT. The OXRAY system addresses these limitations by expanding the field size to 30 cm, enabling 6-MV FFF delivery, adopting 2.5-mm MLCs and, importantly, incorporating VMAT capability for DTT. The details of this system are described elsewhere [10–12].

A man in his 40s with Budd-Chiari syndrome had been diagnosed with HCC 10 years earlier. He was initially treated with transcatheter arterial chemoembolization for a lesion in segment 5, followed by SBRT using DTT-IMRT (60 Gy in 15 fractions) after fiducial marker (VISICOIL, IBA Dosimetry, Belgium) implantation for treatment of the residual tumor. During follow-up, the patient developed four recurrences outside the previously irradiated region, all of which were treated with radiofrequency ablation (RFA). Nine and a half years after the initial treatment, gadoxetic acid-enhanced magnetic resonance imaging (EOB-MRI) revealed recurrence in S5/8 near the portal vein, unsuitable for RFA. Radiotherapy was selected after a multidisciplinary team discussion. The patient was working and preferred a shorter treatment time.

As fluoroscopy showed 20.8 mm of 3D tumor motion, confirming the need for respiratory motion management, this patient was selected for the first implementation of DTT-VMAT for HCC to reduce treatment time for this employed patient. For radiotherapy treatment planning, computed tomography (CT) included breath-hold and 4D-CT scans was acquired with a 64-slice scanner (1 mm slice thickness). The fiducial marker implanted 10 years earlier remained intact and visible on the CT images. The clinical target volume was defined as the gross tumor volume, with a 5-mm isotropic margin and a 6-mm longitudinal margin added to generate the planning target volume (PTV), accounting for setup and tracking error [13]. Due to proximity to the previously irradiated area, the prescribed dose was set to 35 Gy in five fractions, specified as D_90%_ of the PTV. The maximum dose was targeted at approximately 50 Gy, corresponding to the 70% isodose line, when a 100% dose was prescribed to the isocenter.

A single half-arc VMAT plan was created using a RayStation 2023 B (RaySearch Laboratories, Stockholm, Sweden). During treatment, kilovoltage fluoroscopy at 1 Hz verified tracking accuracy. Parameters for 4D modelling and monitoring (125 kV, 320 mA, 10 ms) were optimized daily and fixed regardless of gantry rotation. Intrafractional tracking accuracy was evaluated based on the treatment log files.

Ethics approval was not required because the OXRAY system with DTT is a commercially available device approved by the Pharmaceuticals and Medical Devices Agency, Japan. Written informed consent was obtained from the patient before the SBRT procedure and for the publication of clinical data.

## RESULTS AND DISCUSSION

The scheduled treatment was successfully completed using a previously implanted fiducial marker as a surrogate for the tumor. The peak-to-peak amplitudes of tumor motion due to respiration ranged from 1.8 to 4.5 mm laterally, 11.3 to 20.3 mm longitudinally and 4.5 to 8.5 mm vertically. The associated 4D modelling errors had mean values within 0.4 mm in all directions for all fractions except for fraction 5, for which the mean errors were 0.7 mm laterally, 1.0 mm longitudinally and 1.8 mm vertically; all were well within clinically desirable limits, indicating high geometric accuracy. Details of tumor motion and 4D modelling errors are shown in Table 1. The mean treatment time, including patient setup, 4D model construction and beam delivery, was 23.2 min (range, 12–35 min) across all five fractions. No updates to the 4D model were made during beam delivery. Based on log file analysis, the average 3D tracking error over five treatment days was 2.4 ± 1.8 mm overall, comprising 0.3 ± 1.0 mm laterally, −1.5 ± 2.2 mm longitudinally and −0.1 ± 1.0 mm vertically (Table 2).

**Table 1:** Peak-to-peak tumor motion due to respiration and the associated 4D modelling error

	Lateral [mm]		Longitudinal [mm]		Vertical [mm]	
	Peak-to-peak	4D modelling error	Peak-to-peak	4D modelling error	Peak-to-peak	4D modelling error
Fraction 1	3.7	0.1 ± 0.2	17.2	0.3 ± 0.4	8.1	0.1 ± 0.1
Fraction 2	3.1	0.2 ± 0.2	16.5	0.2 ± 0.3	6.3	0.1 ± 0.1
Fraction 3	1.8	0.2 ± 0.2	11.4	0.3 ± 0.3	6.3	0.2 ± 0.2
Fraction 4	4.5	0.2 ± 0.2	20.3	0.4 ± 0.5	8.5	0.3 ± 0.3
Fraction 5	4.0	0.7 ± 1.8	14.4	1.0 ± 2.3	4.5	1.8 ± 4.5

The 4D modelling errors are presented as mean ± standard deviation.

**Table 2:** 3D tracking errors and detection rate

	Fraction 1	Fraction 2	Fraction 3	Fraction 4	Fraction 5	Total
3D tracking error (mm)	3.0 ± 1.5	1.3 ± 0.8	1.5 ± 0.6	2.1 ± 2.7	3.9 ± 1.1	2.4 ± 1.8
Lateral (mm)	−2.1 ± 1.7	0.3 ± 0.5	0.6 ± 0.6	−0.3 ± 0.5	0.4 ± 0.6	0.3 ± 1.0
Longitudinal (mm)	1.2 ± 1.1	−0.5 ± 0.8	0.8 ± 0.9	−1.8 ± 2.5	−3.7 ± 1.0	−1.5 ± 2.2
Vertical (mm)	−0.4 ± 1.2	−0.8 ± 0.6	0.5 ± 0.5	0.4 ± 1.1	−0.4 ± 0.8	−0.1 ± 1.0
Total number of acquired images	124	125	125	99	99	570
Detected number of acquired images	111	84	95	88	92	470
% of detected	89.5%	67.2%	76.0%	88.9%	92.9%	82.5%

Positive directions of the coordinate system were defined as lateral, longitudinal and vertical, respectively. The 3D tracking errors are presented as mean ± standard deviation.

**Fig. 1:**
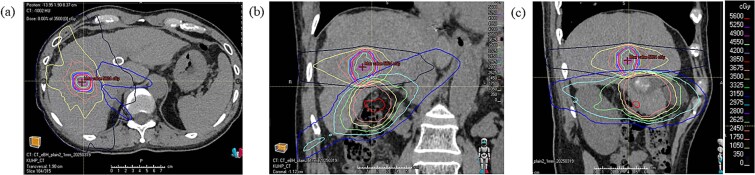
Dose distributions of stereotactic body radiotherapy (SBRT) with dynamic tumour tracking (DTT)-volumetric modulated arc therapy (VMAT) shown in axial (a), coronal (b), and sagittal (c) slices. The isodose lines of the previous irradiation are displayed as follows: 60 Gy (red), 50 Gy (orange), 40 Gy (yellow), 30 Gy (light green), 20 Gy (cyan), and 10 Gy (blue).

The cumulative mean liver dose was 12.3 Gy (calculated as nominal doses; Fig. 1), with a liver volume of 1142.1 cm^3^. Although the current and previous PTVs did not overlap, the minimum distance between them was 5.0 mm. In the region overlapping the previous irradiation field, the cumulative maximum point dose and D_0.03cc_ were 94.0 Gy and 92.8 Gy, respectively (both nominal doses). The cumulative maximum doses delivered to regions previously irradiated with ≥60 Gy, 50 Gy, 40 Gy, 30 Gy, 20 Gy and 10 Gy were 2.3 Gy, 11.9 Gy, 27.2 Gy, 41.5 Gy, 45.6 Gy and 46.9 Gy, respectively. The spared liver volume receiving <15 Gy was 813.9 cm^3^, which meets commonly accepted dose constraints [14].

In this first clinical implementation, gimbal-based DTT-VMAT enabled efficient delivery despite more than 20 mm of 3D respiratory motion. The treatment time was shorter than those reported for DTT-3D-CRT in HCC (mean, 28 min) [4] and for DTT-IMRT in pancreatic cancer (mean, 24.5 min) [15], where daily doses are lower than those for HCC, indicating that separating real-time tumor tracking from VMAT can reduce overall treatment time even in a re-irradiation setting. Robust respiratory motion management is essential because intra- and inter-fractional variations and baseline drift can occur during SBRT in thoracic or abdominal tumors [16–19]; however, in this case gimbal-based DTT-VMAT allowed completion of the planned treatment without additional 4D model updates, while maintaining tracking performance comparable to that reported for the Vero4DRT system, with modelling errors within approximately 1 mm [20]. These findings suggest that gimbal-based DTT-VMAT is advantageous among available respiratory motion management approaches, as it shortens treatment time, mitigates baseline drift and improves patient compliance.

We had certain limitations. First, the number of image acquisitions varied across the five fractions, because beam interruptions require gantry repositioning and image reacquisition. These issues are expected to stabilize through the software and system upgrades planned for future versions. Second, the 3D tracking errors, defined as the difference between the detected fiducial marker and the predicted tumor position, were generally comparable to those in a previous SBRT report with DTT-3D-CRT, except in the longitudinal direction [4]. This may be improved by enhancing the image quality and algorithm refinement. Furthermore, safety, efficacy and optimal indications for this technique will be clarified in the ongoing trials (trial number: jRCTs052240112).

In summary, we report the first clinical implementation and completion of SBRT with DTT-VMAT using a gimbal-mounted linac, the OXRAY system, achieving a mean treatment time of 23.2 min, while maintaining a high tracking accuracy of 2.4 mm. Further studies with larger cohorts are warranted to establish its safety and efficacy.

## Presentation at conference

Part of this work was presented as a poster at the 38th Annual Meeting of the Japanese Society of Radiation Oncology (JASTRO).
